# Molecular cloning, modeling and differential expression of a gene encoding a silent information regulator-like protein from *Sporothrix schenckii*

**DOI:** 10.3892/ijmm.2014.1719

**Published:** 2014-03-31

**Authors:** BINBIN HOU, XIAOMING LIU, FANGLIANG ZHENG, XUEZHU XU, ZHENYING ZHANG

**Affiliations:** 1 Department of Dermatology, The Second Affiliated Hospital of Dalian Medical University, Dalian, Liaoning, P.R. China; 2 Department of Dermatology, Hong Kong University - Shenzhen Hospital, Shenzhen, Guangdong, P.R. China; 3 Department of Dermatology, The First Affiliated Hospital of Dalian Medical University, Dalian, Liaoning, P.R. China; 4 Key Laboratory of Animal Resource and Epidemic Disease Prevention, Life Science School of Liaoning University, Shenyang, Liaoning, P.R. China

**Keywords:** SsSir2, *Sporothrix schenckii*, gene cloning, molecular modeling, differential expression

## Abstract

*Sporothrix schenckii* (*S. schenckii*) is a dimorphic fungus that produces lymphocutaneous lesions. The signature characteristic of *S. schenckii* is a temperature-induced phase transition. Silent information regulator (Sir) has been proven to be involved in phenotypic switching in *Saccharomyces cerevisiae* (*S. cerevisiae*) and *Candida albicans (C. albicans)* by organizing chromatin structure. In this study, we isolated and characterized a Sir homologue gene, designated as SsSir2, from the yeast form of *S. schenckii*. The full-length SsSir2 cDNA sequence is 1753 bp in size and contains an open reading frame of 1329 bp encoding 442 amino acids. The predicted molecular mass of SsSir2 is 48.1 kDa with an estimated theoretical isoelectric point of 4.6. The SsSir2 kinase domain shows a 78% identity with that of Hst2, a Sir2 Ib gene from *S. cerevisiae*. Three exons and two introns were identified within the 1472-bp SsSir2 genomic DNA sequence of *S. schenckii*. A three-dimensional model of SsSir2 was constructed using a homology modeling method, and its reliability was evaluated. The active site of SsSir2 was identified by docking simulation, which indicated that several important residues, such as Asn127 and Asp129, play an important role in the histone deacetylase activity of Sir2 family proteins. The differential expression of the SsSir2 in two stages was demonstrated by real-time RT-PCR. The expression of SsSir2 was higher in the yeast stage compared with that in the mycelial one, which indicated that SsSir2 may be involved in the phenotypic switching and morphogenesis of the yeast phase in *S. schenckii*.

## Introduction

*Sporothrix schenckii (S. schenckii)*, the causative agent of sporotrichosis, is a dimorphic fungus that produces lymphocutaneous lesions. The signature characteristic of *S. Schenckii* is a temperature-induced phase transition. *S. schenckii* grows as a mold in soil at an ambient temperature and is converted to a yeast form after the spores infect the skin of a mammalian host by direct inoculation (1). Although the molecular basis of phenotypic switching in a dimorphic fungus is not well understood, several possible mechanisms have been considered, including genomic rearrangements through epigenetic mechanisms (2). Heritable epigenetic changes are well characterized in *Saccharomyces cerevisiae (S. cerevisiae)*. Regulatory genes that control colony morphology are located in chromosomal positions that exist in two alternative states: a silenced state and an active state. The transition between the two states results from changes in chromatin structure. Phenotypic switching in *S. cerevisiae* occurs when the chromatin state spontaneously changes, a characteristic of silenced domains (3). The silent information regulator (Sir) genes are required to establish the silent state, and mutations in these genes can affect the efficiency with which *S. cerevisiae* cells pass on the silent state to their daughter cells (4). Phenotypic switching in *Candida albicans (C. albicans)* could in principle be controlled using a similar mechanism (5). In a recent study of ours, differentially expressed proteins altered by phenotypic switching in *S. schenckii* were identified using two-dimensional electrophoresis. One of these proteins is homologous to the Sir2 protein from *Aspergillus fumigatus* and its expression increases in the early yeast form of *S. schenckii* (6).

In this study, we describe the molecular cloning of the *S. schenckii* Sir homologue, designated as SsSir2. We performed functional analysis of the SsSir2 gene, and detected differential gene expression in the dimorphic switch of *S. schenckii*. The data presented in this study may broaden our understanding of the function of the SsSir2 gene from *S. Schenckii*.

## Materials and methods

### Fungal strain, media and growth conditions

The strain of *S. schenckii* which was used, ATCC10268, was maintained at the Research Center for Pathogenic Fungi, Dalian Medical University, Dalian, China. To obtain a mycelial culture, the ATCC10268 isolate was inoculated on Sabouraud dextrose agar (SDA) medium (Sigma, St. Louis, MO, USA) and incubated at 25°C. The mycelial colonies thus obtained were inoculated in Sabouraud fluid medium (Sigma) and cultured with shaking at 100 rpm at 25°C for 96 h. To achieve the switch of *S. schenckii* from the mycelial phase to the yeast phase, mycelial colonies were transferred to brain heart infusion (BHI) liquid medium at 37°C and shaken at 100 rpm for 96 h. The mycelial and yeast pellets were collected by centrifugation and stored at −80°C.

### Total RNA, genomic DNA isolation and gene cloning

Approximately 100 mg samples of *S. schenckii* in the mycelial and yeast phase were separately pulverized under liquid nitrogen with a mortar and pestle. Total RNA isolation was carried out according to the manufacturer’s instructions using TRIzol reagent (Invitrogen Life Technologies, Carlsbad, CA, USA) and treated with the RNase-free DNase I kit from Takara (Tokyo, Japan) to eliminate DNA contamination. Genomic DNA was isolated from the yeast phase colonies following the manufacturer’s instructions using the InstaGene™ Matrix kit (Bio-Rad Laboratories, Hercules, CA, USA). cDNA was synthesized from 500 *μ*g of total RNA of ATCC10268 by murine leukemia virus reverse transcriptase (MLV-RT) (Takara) primed with oligo(dT) following the manufacturer’s instructions, and used as a template for PCR. Degenerate primers, SsSir2-F1 and SsSir2-R1, were designed based on multiple alignments of the highly conserved Sir2 domains of *C. albicans* Sir2 (NW139502), *Cryptococcus neoformans* Sir2 (NC009183) and *Ustilago maydis* Sir2 (XM756946) amino acid sequences. The PCR product of expected size was cloned into the pMD18 vector (Takara) and sequenced. The degenerate primers yielded a 248 bp fragment homologous to known Sir2. To obtain the full-length cDNA sequence of the SsSir2 gene, 5′-RACE and 3′-RACE experiments were performed using the 5′-Full RACE kit and 3′-Full RACE Core Set Ver. 2.0 kit (Takara) according to the manufacturer’s instructions. Nested PCR was then performed. Briefly, five specific primers, 3′SIR2-1 and 3′SIR2-2 of 3′-RACE and 5′SIR2-1, 5′SIR2-2 and 5′SIR2-3 of 5′-RACE, were synthesized based on the cDNA sequence obtained by the degenerate primers. PCR products of 5′-RACE and 3′-RACE were both cloned into the pMD18 vector (Takara) and sequenced. To determine the nucleotide sequence of the genomic DNA corresponding to SsSir2, PCR was performed using the primers, SsSir2-G1 and SsSir2-G2, and genomic DNA as a template. The PCR products were then sequenced. The sequences of all the primers used in this study are listed in Table I.

### Bioinformatic and phylogenetic analysis of SsSir2

The nucleotide sequences were analyzed using Sequence Scanner software (Applied Biosystems/Life Technologies Corp., Carlsbad, CA, USA) and the BLAST network service of the National Center for Biotechnology Information (NCBI) (http://www.ncbi.nlm.nih.gov/blast). The open reading frame was found by the ORF Finder (http://www.ncbi.nlm.nih.gov/gorf/gorf.html). For the exact localization of the exon/intron boundaries the mRNA-to-genomic alignment program, Spidey (http://www.ncbi.nlm.nih.gov/IEB/Research/Ostell/Spidey/index.html), was used. The deduced amino acid sequence was analyzed with the Expert Protein Analysis System (http://www.expasy.org/) and the protein domain characteristics of SsSir2 were determined using the Simple Modular Architecture Research Tool (http://hits.isb-sib.ch/cgi-bin/PFSCAN). Isoelectric point and molecular weight prediction were carried out at (http://cn.expasy.org/tools/pi_tool.html). Multiple alignments of SsSir2 were performed with the ClustalW Multiple Alignment Program (http://www.ebi.ac.uk/clustalw/).

### Molecular modeling

The SsSir2 sequence was searched for a similar sequence using the PSI-BLAST against Protein Data Bank (PDB). The PSI-BLAST results yielded the X-ray structure of the human histone deacetylase SIRT2 (PDB code: 1J8F) with a 47.7% identity to our SsSir2 protein. The fold recognition server, pGenTHREADER, identified SIRT2 as a good template with a good confidence level. Consequently, the crystal structure of SIRT2 was used as the template for modeling SsSir2. The amino acid sequence of SsSir2 was aligned with the sequence of SIRT2 using the align 2D module in MODELLER. Manual inspection and modification of the alignment was performed after initial model generation and analysis of the DOPE score profile of the generated model. Good sequence alignment was obtained for 280 amino acids of the SsSir2 sequence; however the first 17 and the last 145 amino acids were not presented in the alignment. Homology models were built based upon the alignment of the sequence using the automated homology modeling program, MODELLER 9v9, with default parameters. Loops were refined using the loop-model module of MODELLER with a fast molecular dynamics. The best model was selected from 3,000 candidates based on the evaluation of PDF total energy, verify score and Ramachandran plot. Subsequently, to remove bad contacts between side-chains, the best model was refined by a 3,000 step energy minimization with a constraint on backbone atoms using the molecular dynamics simulation program, NAMD. The quality of the final model was examined by Verify3D, ERRAT and the Ramachandran plot.

### Molecular docking

Molecular docking can fit molecules together in a favorable configuration to form a complex system. The structural information from the theoretically modeled complex may help us to clarify the binding mechanism between SsSir2 and NAD. In order to gain insight into the binding mode of SsSir2 with NAD, the automated docking program, AutoDock v4.2, was used to perform the molecular docking. AutoDock uses a Lamarckian genetic algorithm to explore the binding possibilities of a ligand in a binding pocket. This program allows the ligand to be flexible, whereas the protein side-chains remain fixed. For our docking experiment, a grid of 80×70×70 points was generated with a grid SIR2-R1 spacing of 0.375 Å around the large groove which was supposed to be the NAD binding site. This grid space defines the region of the protein in which the ligand searched for the most favorable interactions. A total of 200 dockings were carried out with 27,000 cycles per run; interaction energies were calculated for various docked positions and ranked in accordance with the interaction energies between the ligand and the protein. Finally, the docked complex of SsSir2 and NAD was selected according to the criteria of the hydrogen bond network combined with the interaction energy.

### Differential expression of SsSir2 in two stages during the dimorphic switch

The expression of the SsSir2 transcript in the different stages (mycelial and yeast) were measured by real-time RT-PCR. Primers and a TaqMan probe for target genes were designed with PrimerSelect in the Lasergene software package (DNAStar Inc., Madison, WI, USA) and are listed in Table I (24T, 8F, 58R). Fifty nanograms of total RNA were assayed from two stages of *S. schenckii* in triplicate using the PrimeScript RT-PCR kit (Takara). The minus-reverse transcriptase control was also performed in triplicate. The amplification conditions were optimized for the ABI PRISM 7500 instrument (Applied Biosystems, Carlsbad, CA, USA). The cycling conditions using TaqMan probe detection were as follows: 95°C for 2 min followed by 40 cycles at 95°C for 10 sec, 61°C for 10 sec, 72°C for 40 sec. 18srDNA was selected as the endogenous control. The relative quantification of target gene expression was carried out using the comparative cycle threshold (CT) method as previously described by Livak and Schmittgen (7). The ΔCT value was determined by subtracting the target CT value of each sample from its respective 18srDNA CT value. The calculation of ΔΔCT involved using the mycelial sample ΔCT value as an arbitrary constant to subtract from yeast sample ΔCT values. Differences in the expression of target genes were determined by 2−ΔΔCT. Data are expressed as arithmetic means ± SD unless otherwise indicated. A comparison between mycelial and yeast samples was performed using the Student’s t-test. Differences with a P-value of <0.05 were considered to be statistically significant.

## Results

### Cloning and sequence analysis of SsSir2

**Figure 1:**
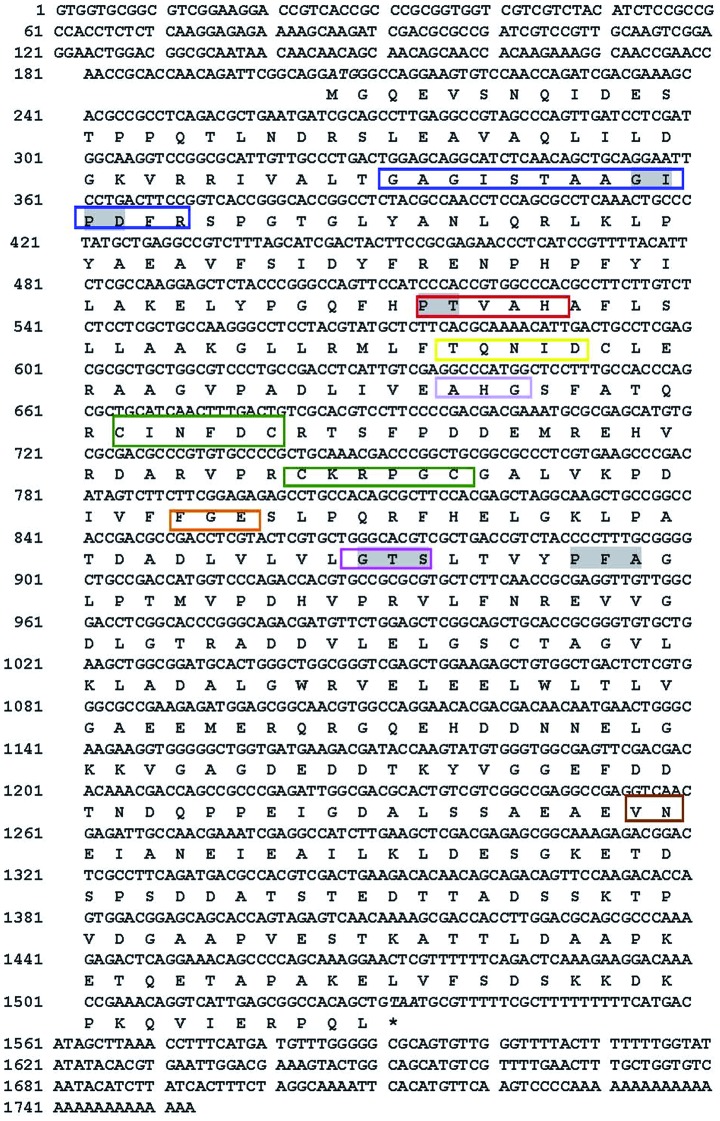
Nucleotide sequence of SsSir and the predicted amino acid sequence of the SsSir2 product. The cDNA sequence of SsSir2 is listed in the top lines. Deduced amino acid sequence of the open reading frame (ORF) is shown by the single-letter amino acid codes. Several short motifs of conserved amino acids, including GAGISXXXGIPXXR (blue), PXXXH (red), TQNID (yellow), AHG (purple), two sets of CXXXXC (green), FGE (orange), GTS (pink) and VN (brown), are shown in different color shades. Both GIPD in the GAGISXXXGIPXXR motif and PT in the PXXXH motif are shown in underlined texts. PFA locating five residues C-terminal to the GTS motif is also shown in underlined text.

A full-length SsSir2 cDNA (1753 bp) including an open reading frame of 1329 bp, encoding 442 amino residues, was flanked by a 204 bp 5′-untranslated region (5′-UTR) and a 220 bp 3′-UTR (Fig. 1). The SsSir2 genomic DNA is 1472 bp in length. The aligned results revealed that there were two introns between the sequences of the genomic DNA and the cDNA. Its 5′ and 3′ ends conformed to the basic consensus, GT/AG, for the eukaryotic splice donor and acceptor site. Based on the sequence of the cDNA, the molecular weight of the predicted amino acid is approximately 48.1 kDa, the theoretical pI is 4.6. Motif searches and sequence comparison revealed that SsSir2 consists of both a 187-amino acid conserved enzymatic core domain (residues 43–229) and extended N- and C-terminal sequences usually present in eukaryotic Sir2 homologues (Fig. 1). Database searches revealed that there are several short motifs of conserved amino acids present in the enzymatic core domain of SsSir2; these include GAGISXXXGIPXXR, PXXXH, TQNID, HG, two sets of CXXXXC that may be a zinc-finger motif, FGE, GTS and VN (Fig. 1).

### Homology and phylogenetic analysis of SsSir2

**Figure 2:**
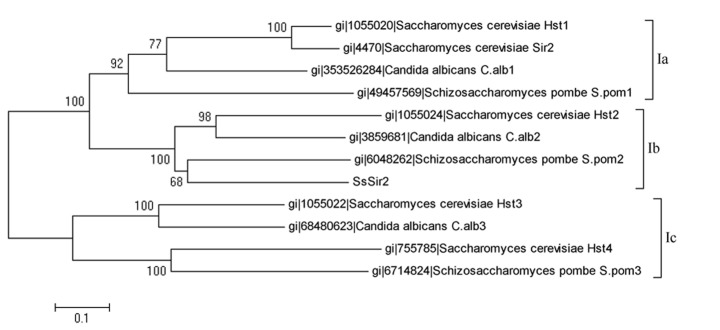
Phylogenetic association among class I sirtuins proteins. An NJ tree was generated by MEGA Ver. 5.05 with bootstrap analysis based on 500 replications. Percentage bootstrap values are shown at branch points. Scale bar indicates the number of substitutions per site. SsSir is a member of class Ib sirtuins proteins.

**Figure 3:**
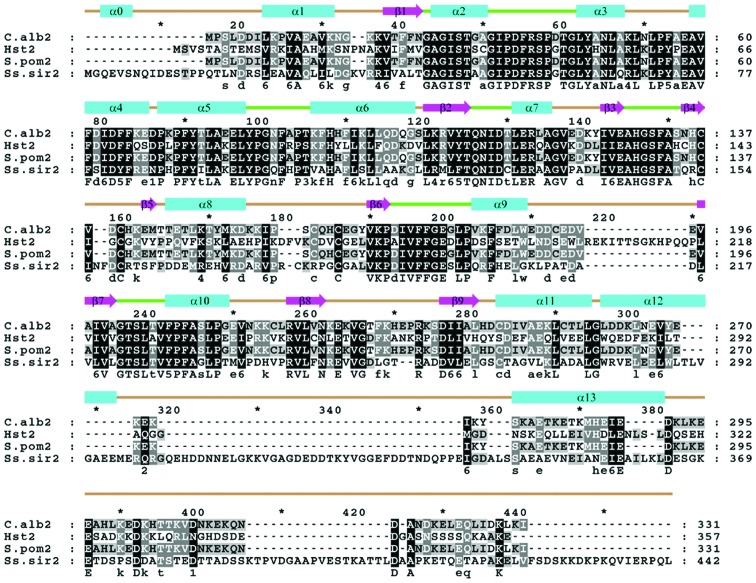
Multiple sequence alignment of SsSir to other fungal Sir2 of class Ib sirtuins. The amino acid sequence of C. alb2 [*Candida albicans* (*C. albicans*), CAA22018], Hst2 [*Saccharomyces cerevisiae* (*S. cerevisiae*), U39063] and S. pom2 (Schizosaccharomyces pombe, AL121807) were aligned using the online version of ClustalW. Shade residues indicate ≥75% homology (black) or ≥50% homology (gray). The positions of α helices and β strands are indicated above the sequence. α helices are presented in blue boxes, β strands are presented in purple boxes with an arrow, and loops are presented in brown lines, except for the Sir2 conserved loops which are highlighted in green lines.

To clarify the association between SsSir2 and other Sir2-like proteins, we calculated multiple sequence alignments of these sequences. The derived evolutionary tree is split into two main branches, one formed by some class Ia and class Ic sirtuins, the other one by class Ib sirtuins, including the SsSir2 sequence (Fig. 2). Further alignment clearly revealed that the SsSir2 kinase core domain is highly homologous to that of other class Ib sirtuins, such as Hst2 (U39063), C.alb2 (CAA22018 ) and S.pom2 (AL121807) (Fig. 3). These proteins share a similar structure and have 78, 80 and 75% amino acid similarities in the core domain, respectively. These proteins have a highly conserved catalytic core domain in common, but display a variation in their extended N- and C-terminal domains, which may have a function in target specificity of Sir2, as mutations outside the enzymatic core domain of the yeast Sir2 selectively affect distinct forms of silencing.

### Three-dimensional (3D) model of SsSir2 protein

**Figure 4:**
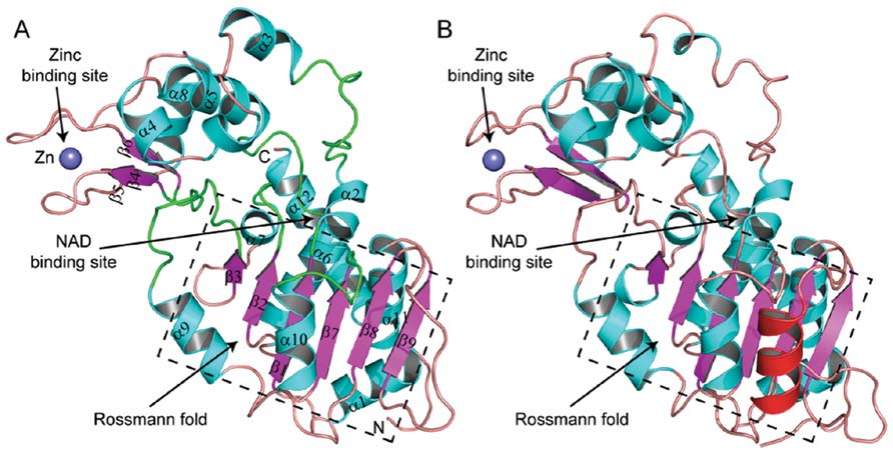

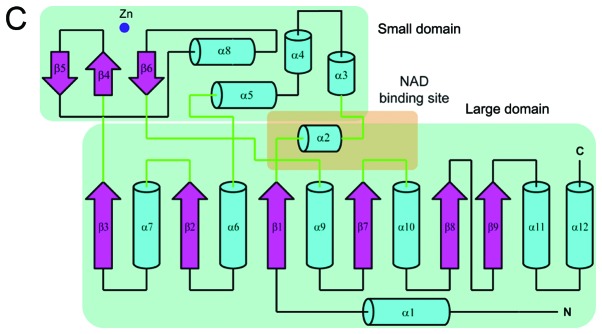
Overall three dimensional (3D) structure models of (A) SsSir protein and (B) human histone deacetylase SIRT2. Blue ribbons are α-helices and purple ribbons are β-strands. The SsSir2 conserved loops are colored green in the left panel. The SsSir2 absent helix is colored red. Zinc ribbon motifs are present in the small domains. Both SsSir2 and SIRT2 include two domains: a small domain and a larger domain (forming Rossmann fold). The zinc ions are shown as blue balls. The NAD^+^ binding sites are located at the junction of the two domains. (C) Topological diagrams of secondary structure elements. Small, large domains and NAD, zinc binding sites are all indicated.

**Figure 5:**
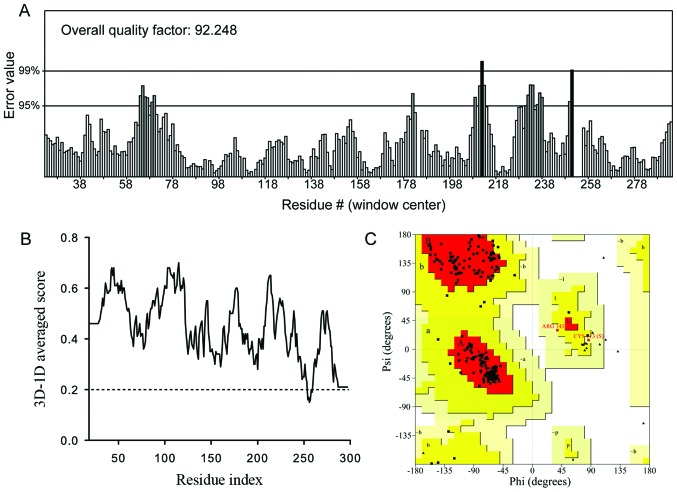
Evaluation of the SsSir three dimensional (3D) structure by (A) ERRAT, (B) Verify 3D and (C) Ramachandran plot.

The homology model of SsSir2 was constructed based on the X-ray crystal structure of human histone deacetylase SIRT2 (PDB code: 1J8F), which had relatively high sequence identity (47.7%) with SsSir2, and the manually modified sequence alignment of SsSir2 and SIRT2. Following the homology modeling and loop refinement, the best model was selected from 3,000 candidates. The final model (Fig. 4A) was then obtained after several rounds of energy minimization to remove bad contacts between side-chains, and its quality was further validated by ERRAT, Verify3D and the Ramachandran plot (Fig. 5). The non-bonded interaction between different atoms was analyzed by ERRAT. The ERRAT overall quality factor for the SsSir2 model was 92.248 (Fig. 5A), which was within the range of a high quality model. The Verify3D results (Fig. 5B) for the SsSir2 model revealed that 98.22% of the residues had an averaged 3D-1D score of >0.2, which indicates a well built model since almost all the residues are valid in their folded conformation. The Ramachandran plot (Fig. 5C) showed that for the SsSir2 model, 92.4% residues were in most favored regions, 6.7% in the additional allowed regions, 0.8% in the generously allowed regions and none of the residues were in the disallowed region, which means that this model has a high stereochemical quality.

The SsSir2 model contains 280 amino acids of the SsSir2 sequence, and the first 17 and the last 145 amino acids were not built, as these regions were predicted to be unstructured or loosely folded. SsSir2 includes two domains: a larger domain that consists of 8 α helix and 6 parallel β sheets and forms a variant of the Rossmann fold, which present in many NAD(H)/NADP(H) binding enzymes as prosthetic group, and a small domain that consists of 4 α helix and 3 anti-parallel β sheets and a zinc atom tetrahedrally coordinated by four cysteine residues (Figs. 3 and 4A and C). The two domains are connected by four loops (51–61, 98–105, 145–151 and 192–201) (Fig. 4A and C), which are highly conserved in the Sir2 protein family (Fig. 3). These four loops and other three conserved loops (43, 126–130 and 222–227) of the large domain form a large groove, which was supposed to be the NAD binding site (Fig. 4A and C). By comparing the structure of SsSir2 and SIRT2, we found that the two structures were exactly similar, particularly in the NAD binding site, zinc-binding site and the Rossmann fold, although the amino acids are not so conserved in these regions. The distinct difference between SsSir2 and SIRT2 was that the α helix that connects the fifth (β8) and sixth (β9) β sheets of the Rossmann fold was absent (Fig. 4A and B).

### Molecular interaction analysis between SsSir2 and NAD

**Figure 6:**
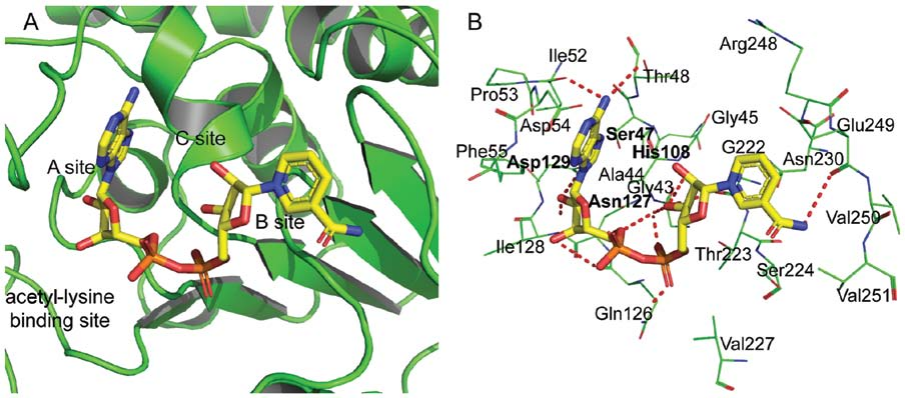
The complex and detailed binding mode of SsSir with NAD. A ribbon diagram showing the NAD binding pocket in the open conformation. Three distinct regions inside the pocket, designated A, B and C sites are indicated. (A) Key residues discussed in the text are shown in a ball-and-stick model. The NAD molecule is shown in a magenta stick model. (B) Hydrogen bonds are indicated with red dashed lines.

To further understand the interaction between SsSir2 and its prosthetic group, NAD was docked into the NAD binding site of SsSir2 by molecular docking. The optimal binding conformation of the SsSir2-NAD complex was selected and is presented in Fig. 6. The NAD molecule is bound in a large groove formed by the conserved loops between the large and small domain of SsSir2. The NAD binding pocket can be divided into three regions (Fig. 6A): i) site A, where the adenine-ribose moiety of NAD is bound; ii) site B, where the nicotinamide-ribose moiety is bound; and iii) site C, which is deep inside the pocket and is not near the position of observed NAD. The detailed interacting residues and hydrogen bonds are both labeled in Fig. 6B.

### Expression of SsSir2 in two stages of S. schenckii

The mRNA expression of SsSir2 in the different stages was analyzed by real-time RT-PCR normalized against 18srDNA levels. The expression was determined as fold increased 2^−ΔΔCt^ levels relative to the stage with the lowest expression (mycelial) set to 1. The SsSir2 gene was expressed in two stages of *S. schenckii*, with higher mRNA levels observed in the yeast form (1.72-fold). There were significant differences observed between the mycelial and yeast form (Table II).

### Accession number

The full length of the cDNA sequence and genomic DNA sequence of the SsSir2 gene were submitted to the GenBank database under the accession number (JX312330) and (KC304788), respectively.

## Discussion

The Sir2-like protein is the founding member of a large family of NAD^+^-dependent deacetylases, the so-called sirtuins, conserved from bacteria to mammals (8). Sirtuins were grouped into five main branches designated as classes I–V. Specific amino acid sequence motifs characterize the different classes of sirtuins. In class III sirtuins, the GAGISXXXGIPXXR motif is usually GAGISAESGIPTFR, whereas in class II, it is GAGISTESGIPDYR. Sirtuins of classes I, IV and V also have GIPD within this motif. In sequences of classes II, III and U, the PXXXH motif is PNXXH, while in the eukaryotic class I and IV sequences a T or S usually follows the P. The HG motif is strictly conserved in all known sirtuins. In class Ia the HG motif reads CHG, in class Ib it reads AHG and in class Ic (and in most non-class I sirtuins) it reads LHG. The three residues located five residues C-terminal to the GTS motif are rather useful in differentiating between the types of sirtuins. The three residues usually observed at this position for each class and subclass are: Ia (PVA or PVS), Ib (PFA), Ic (GVK), V (PAA), II (SGY), III (PAA), IVa (PXX) and IVb (KKY) (9). In the present study, SsSir2 was labeled as a class I sirtuin based on the presence of GIPD in the GAGISXXXGIPXXR motif and PT in the PXXXH motif (Fig. 1). We further identified SsSir2 as a class Ib sirtuins for its AHG sequence and PFA residues located five residues C-terminal to the GTS motif, which was also confirmed by phylogenetic association analysis.

In the absence of an experimentally determined crystal structure, it is generally recognized that the homology modeling of proteins is currently the most accurate method for 3D structure prediction (10). Homology modeling is based on the assumption that the proteins with similar sequences might have analogous 3D structures. Thus, the selection of a suitable template is the first step. In the present study, SIRT2 was selected as the template for constructing the 3D model of SsSir2 as it has a 47.7% amino acid sequence identity with SsSir2. To assess whether the 3D model of SsSir2 is of reasonable quality, ERRAT, Verify3D and the Ramachandran plot were used and showed positive results (11). The results of quality assessment suggested that the model of the SsSir2 structure was of reasonable quality compared to the crystal structure of the SIRT2 and sufficient for use in further experiments.

The NAD molecule is bound in an extensive pocket between the large and the small domains of the SsSir2 protein (12). Several important residues, such as Asn127 and Asp129 identified by the current docking simulation, have been shown to play an important role in the histone deacetylase activity of Sir2 family proteins. His108 and Asn127 were shown to guide the substrate to the correct position with catalytic activity in the catalytic reaction. The other two conserved amino acids of Ser47 and Asp129 at the bottom of the protein stabilize the adenine ribose and adenine of NAD^+^ which combined with SsSir2, but did not interact directly with NAD^+^. These characters of crystal structure are similar to those reported in the study by Min *et al* (12). In our study, as shown in Fig. 6B, several hydrogen bonds, which are a key interaction force of protein ligand binding, were also found between NAD and some important conserved residues. All of the above properties indicate that the predicted binding mode was reliable and that the complex was stable.

Sir2 has been proven to be involved in morphogenesis and phenotypic switching in dimorphic fungi. Rine and Herskowitz established that sporulation in *S. cerevisiae* requires the functional gene products of Sir2 (13). A screen for *S. cerevisiae* temperature-sensitive silencing mutants identified a strain with a point mutation in the Sir2 gene (Ser276 was changed to Cys). Haploid strains carrying the mutation were severely defective at mating at 37°C but normal at 25°C (14). In *C. albicans*, the deletion of the Sir2 gene produces a dramatic phenotype: variant colony morphologies arise at frequencies as high as 1 in 10. The morphologies resemble those described as part of a phenotypic switching system proposed to contribute to pathogenesis (4). In *S. schenckii*, the germination of conidia is a key pathogenicity determinant, as conidia are infectious propagules. The question remains of whether SsSir2 has the same function in the phenotypic switching of *S. schenckii* similar to the Sir2 ortholog in other dimorphic fungi. In our previous study, the SsSir2 protein expression level in yeast cells was found to be 5.47-fold higher than that in the mycelial phase of *S. schenckii* (6). In this study, the mRNA expression of SsSte20 in yeast cells of both ATCC10268 and a clinical *S. schenckii* isolate from a patient with fixed sporotrichosis (data not shown) were higher than in the mycelial ones. These results suggest that SsSir2 may also play a role in the morphologic switching of *S. schenckii*, presumably by transcriptional silencing of the silent mating type loci.

A variety of environmental signals may affect the mycelial-yeast transition (15), and a large body of evidence suggests the existence of several signaling pathways (16–19), any one of which may be involved in the mycelial-yeast transition. The ability of *S. schenckii* to switch between different phenotypes is an alternative way to obtain the variability required to survive an uncertain environment. The mechanisms through which the SsSir2 gene controls one or more of these pathways remain to be elucidated. It is most likely that some types of external stress may suppress the Sir2 gene and thereby activate one or more of these signaling pathways which regulate the mycelial-yeast transition when it may prove beneficial. This idea has some support from the discovery that gene silencing in *S. cerevisiae* (which requires the Sir2 gene) is inactivated as cells become older (20). Perhaps a different type of stress would produce a similar inactivation of silencing in *S. schenckii*. The detailed functions of SsSir2 require further investigation. We are currently carrying out further experiments on generating SsSir2 mutants to investigate its detailed functions in this important fungal pathogen.

## Figures and Tables

**Table I:** Sequences of primers used in this study.

Name	Sequence (5′→3′)	Length (bp)
SIR2-F1	ACNGCNGCNGGNATHCCYGAYTT	23
SIR2-R1	CARTCDATRTTYTGRGTRAA	20
3′SIR2-1	GAACCCTCATCCGTTTTACATTCTCGCC	28
3′SIR2-2	ACGCCTTCTTGTCTCTCCTCGCTGCC	26
5′SIR2-1	GAGAGACAAGAAGGCG	16
5′SIR2-2	GAGAATGTAAAACGGATGAGGG	22
5′SIR2-3	AGCATAGGGCAGTTTGAGGCG	21
G1	ATGGGCCAGGAAGTGTCCA	19
G2	GCGGCGGCCGCCAGCTGTGGCCGCTCAATG	30
8F	GGAACTTACAAGACCATCAT	20
58R	GAGTTGGCCACTGGTTT	17
24T	FAM-CAGTGCCTCAAATTCCAA-TAMRA	19

Degenerate primers were designed based on multiple alignments of the highly conserved SIR2 domains for gene cloning: SIR2-F1 and SIR2-R1. Primers for 3′-RACE were 3′SIR2-1 and 3′SIR2-2; and for 5′-RACE were 5′SIR2-1, 5′SIR2-2 and 5′SIR2-3. To determine the nucleotide sequence of the genomic DNA corresponding to SsSIR2, PCR was performed using the primers, G1 and G2. Primers and a TaqMan probe of real-time RT-PCR were used: 8F, 58R and 24T.

**Table II:** Relative abundance of differential gene expression as determined by real-time RT/PCR.

cDNA name	Phase	Target CT	18srDNA CT	ΔCT	ΔΔCT	2 ^−ΔΔCT^
SsSIR2	Mycelial	27.38±0.52	20.66±0.25	6.7±0.37	0±0.37	1
	Yeast	29.59±0.16	22.09±0.60	7.5±0.61	0.79±0.61	1.72

ΔCT, target transcript CT-18srDNA CT normalization of CT for target gene relative to 18srDNA CT. Statistical analysis of normalized expression levels between mycelial and yeast form. Each of the target genes differs significantly (U-test; P<0.05). ΔΔCT, mean yeast ΔCT-mean mycelial ΔCT. The mean value for the mycelial ΔCT was used as a calibrator to set the baseline for comparing mean differences in the ΔCT values of the yeast form. ^2−ΔΔ^CT, normalized target amount relative to the mycelial form. Data are presented as the means ± SD; P<0.01.
